# Sulforaphane Induces Cell Cycle Arrest and Apoptosis in Acute Lymphoblastic Leukemia Cells

**DOI:** 10.1371/journal.pone.0051251

**Published:** 2012-12-12

**Authors:** Koramit Suppipat, Chun Shik Park, Ye Shen, Xiao Zhu, H. Daniel Lacorazza

**Affiliations:** 1 Texas Children’s Cancer and Hematology Centers, Texas Children’s Hospital, Houston, Texas, United States of America; 2 Department of Pathology and Immunology, Baylor College of Medicine, Houston, Texas, United States of America; 3 Summer Medical and Research Training Program, Baylor College of Medicine, Houston, Texas, United States of America; 4 Department of Pediatrics, Baylor College of Medicine, Houston, Texas, United States of America; Institute for Virus Research, Laboratory of Infection and Prevention, Japan

## Abstract

Acute lymphoblastic leukemia (ALL) is the most common hematological cancer in children. Although risk-adaptive therapy, CNS-directed chemotherapy, and supportive care have improved the survival of ALL patients, disease relapse is still the leading cause of cancer-related death in children. Therefore, new drugs are needed as frontline treatments in high-risk disease and as salvage agents in relapsed ALL. In this study, we report that purified sulforaphane, a natural isothiocyanate found in cruciferous vegetables, has anti-leukemic properties in a broad range of ALL cell lines and primary lymphoblasts from pediatric T-ALL and pre-B ALL patients. The treatment of ALL leukemic cells with sulforaphane resulted in dose-dependent apoptosis and G2/M cell cycle arrest, which was associated with the activation of caspases (3, 8, and 9), inactivation of PARP, p53-independent upregulation of p21^CIP1/WAF1^, and inhibition of the Cdc2/Cyclin B1 complex. Interestingly, sulforaphane also inhibited the AKT and mTOR survival pathways in most of the tested cell lines by lowering the levels of both total and phosphorylated proteins. Finally, the administration of sulforaphane to the ALL xenograft models resulted in a reduction of tumor burden, particularly following oral administration, suggesting a potential role as an adjunctive agent to improve the therapeutic response in high-risk ALL patients with activated AKT signaling.

## Introduction

Acute lymphoblastic leukemia (ALL) is the most common hematological malignancy in children. The incidence of the two ALL subtypes correlates inversely with prognosis; approximately 70–80% of ALL cases are of the precursor B-cell lineage (pre-B ALL), whereas T-cell ALL (T-ALL) occurs at a lower frequency and has a worse prognosis [1]. Although risk-adaptive therapy has improved the treatment outcome of childhood ALL, 20% of these patients still relapse after their initial treatment [2]. A poor understanding of ALL pathobiology has prevented the development of targeted therapies to treat relapsed disease, which is the second most common cause of cancer-related deaths in children [3]. Therefore, the development of novel agents is critical to generate frontline therapies for high-risk patients and salvage agents to treat relapsed disease.

Although relapses in pre-B ALL are frequently associated with high-risk disease (i.e., BCR-ABL translocation, hypodiploidy, and MLL rearrangement with slow early response and induction failure), relapses can occur within all currently defined risk groups [3], [4]. The majority of relapses occur in children initially assigned to the standard (i.e., age 1–9 y/o and white blood cells <50,000/µl) or high-risk categories (i.e., age <1y/o or >9 y/o, WBC >50,000/µl, and central nervous system or testicular disease at diagnosis) [3]. The survival rate of patients with relapsed pre-B ALL ranges from 20 to 50%, depending on the site and time of relapse. In contrast, patients with T-cell ALL who develop bone marrow relapse at any time during therapy are more difficult to treat and therefore exhibit worse outcomes compared to relapsed pre-B ALL patients [5], [6]. Thus, a better understanding of the pathogenesis of ALL and the identification of the molecular targets associated with more aggressive disease can aid in the development of new therapeutic strategies for children with high-risk disease. The enhanced ability of leukemic cells to proliferate and survive compared to normal cells is an important factor associated with disease severity [7]. Leukemic cells deregulate proliferation and increase survival through the loss of key cell cycle checkpoint controls, such as CDKN2A/B misexpression, and the activation of pro-survival signals such as the PI3K/AKT/mTOR pathway [8], [9], [10], [11], [12], [13]. Therefore, chemical compounds that are able to induce cell cycle arrest and inhibit survival signals selectively in leukemic cells both *in vitro* and in preclinical mouse models may be the next line of chemodrugs that are utilized to control or eradicate resistant leukemic cells.

Many standard chemotherapeutic agents have been discovered from natural sources (e.g., daunorubicin and cytarabine). Sulforaphane (SF) is a dietary isothiocyanate found in cruciferous vegetables and is endowed with both preventive and therapeutic activities in solid tumors [14]. Epidemiological studies conducted in the US found that individuals who consumed a diet rich in cruciferous vegetables (i.e., broccoli and cabbage) had a lower incidence of breast, lung, prostate, and colon cancer [15], [16], [17], [18], [19]. Furthermore, the consumption of raw cruciferous vegetables inversely correlates with the risk of bladder cancer [20]. This cancer chemopreventive property has been largely attributed to the activity of isothiocyanates derived from the metabolism of glucosinolates that accumulate in cruciferous vegetables. The glucosinolate glucoraphanin is converted to SF by myrosinases that are present in cruciferous vegetables and gut bacteria [19]. SF is a highly reactive and hydrophobic compound that can alter cellular function by entering cells where SF is either conjugated to glutathione and secreted via the mercapturic pathway or it forms thionacyl adducts with the thiol groups of not yet identified proteins in cancer cells. SF induces apoptosis in several cancer cell lines and has been tested in animal models of human cancer, such as T-cell leukemia, breast, colon, and prostate cancer, by targeting caspases, PARP, p21^CIP1/WAF1^ (p21), p53 and Bax [21], [22], [23], [24], [25]. SF also causes G1/S and G2/M cell cycle arrest through alterations in the levels of cyclin A, cyclin B1, cyclin D1, p21^cip1/waf1^, and the tumor suppressor KLF4 [23], [26], [27], [28], [29]. Moreover, a recent report showed that SF restricts the survival of cancer cells by inhibiting PI3K/AKT signaling in the PTEN-null mouse model of prostate cancer [30].

In this study, we describe the anti-leukemic effect of SF in pre-B ALL and T-ALL cell lines and primary samples from pediatric ALL patients. SF induced apoptosis, G2/M cell cycle arrest, higher p21 levels, cleavage of PARP and caspases (3, 8, and 9), and the inhibition of AKT/mTOR signaling in ALL cells. We further confirmed that the administration of SF controlled the expansion of leukemic cells in pre-clinical xenograft models of ALL. This study supports further testing of SF as an adjunctive agent to increase remission rates in high-risk ALL patients and to treat other hematological malignancies with activated survival pathways.

## Materials and Methods

### Ethics Statement

The Baylor College of Medicine Institutional Review Board approved the use of human samples for this study. Primary clinical specimens were obtained from the tissue repository at Texas Children’s Hospital that collected samples following written informed consent. We received archived samples upon approval by the Leukemia Research Interest Group at Texas Children’s Hospital.

All mice were maintained in specific pathogen-free conditions at the Baylor College of Medicine (Houston, TX, USA). All experiments were performed with the approval of the Institutional Animal Care and Usage Committee of Baylor College of Medicine.

### Chemicals

Synthetic DL-Sulforaphane (SF) was purchased from Sigma-Aldrich (St. Louis, MO) and LKT Laboratories (St. Paul, MN) for both the *in vitro* and *in vivo* studies. SF was dissolved in dimethyl sulfoxide (DMSO) to generate a 40 mM stock concentration and stored at −20°C. SF was diluted to the specified final concentration in RPMI-1640 medium supplemented with 10% fetal bovine serum (FBS). The volume of DMSO was less than 0.1% of the total volume, and the identical dilution of DMSO in culture medium was used as the vehicle control. S-(N-Methylsulfinylbutylthiocarbamoyl)-glutathione (SF-glutathione), S-(N-Methylsulfinylbutylthiocarbamoyl)-L-cysteine (SF-cysteine), and N-Acetyl-S-(N-Methylsulfinylbutylthiocarbamoyl)-L-cysteine (SF-NAC) were obtained from LKT Laboratories.

### Cell Lines and Cell Culture

The Nalm-6, REH and RS-4 cell lines were obtained from Dr. Karen Rabin (Texas Children’s Cancer and Hematology Centers, Baylor College of Medicine), originally from the American Type Tissue Collection (REH, RS4) and the DMSZ German Collection of Microorganisms and Cell Culture (Nalm-6). The EBV-transformed lymphoblastoid cell line (LCL) was provided by Dr. Catherine Bollard (Center for Cell and Gene Therapy, Baylor College of Medicine) [31]. The RPMI, DND41 and KOPTK1 T-ALL cell lines were obtained from Dr. Adolfo Ferrando (Columbia University, New York) [32] and Jurkat cells from the American Type Tissue Collection. All of the cell lines were maintained in RPMI-1640 medium (Lonza) supplemented with 10% FBS, 2 mM glutamine, 1 mM sodium pyruvate, penicillin (10 U/ml), and streptomycin (10 µg/ml).

### Patient Samples

Bone marrow or peripheral blood samples from pre-B ALL and T-ALL patients collected at the time of diagnosis were obtained from the Texas Children’s Cancer Center tissue repository (>80% blast cells). The samples were collected after written informed consent and frozen lymphoblasts were supplied in an unspecified manner. All experiments with patient samples conformed to the regulatory standards approved by the Baylor College of Medicine Institutional Review Board (IRB). Mononuclear cells from healthy donors were isolated using Ficoll gradient from peripheral blood collected according institutional review board protocols.

### *In vitro* Cell Viability Assay

The cell lines were cultured overnight in 96-well plates at a density of 2×10^4^ cells/well. The next day, SF was added (0–10 µM), and the cells were incubated for an additional 24–48 hours. The primary patient samples were cultured for 4 hours in 96-well plates at a density of at least 2×10^4^ cells/well followed by an additional 24–48 hours in the presence of SF (0–40 µM). Cell viability was measured using the CellTiter-Glo Luminescent Viability assay (Promega), an ATP-based method, and the number of viable cells was estimated as relative luminescent units. The half-maximal inhibitory concentration (IC50) was calculated from triplicates of each dose by nonlinear regression analysis using GraphPad software.

### Analysis of Apoptosis and DNA Content

The cell lines were cultured in 6-well plates at a final concentration of 4×10^5^ cells/ml and incubated overnight before the addition of SF (7.5 µM). The cells were collected 48 hours later and the percentages of annexin-V- and 7-AAD-positive cells were analyzed by flow cytometry as indicated by the manufacturer (BD Biosciences). To measure DNA content, the cells were resuspended in PBS containing 0.1% sodium citrate, 0.1% Triton-X 100 and RNase A (100 µg/ml), and the nuclei were stained with propidium iodide (50 µg/ml). The cell cycle distribution was analyzed by flow cytometry (FACScanto) and calculated using FlowJo software (Tree Star Inc.).

### Immunoblots

The leukemic cells were harvested after 24 hours of incubation with SF (7.5 µM) or the vehicle control. The cells were lysed with a 1% Nonidet P-40 (NP-40) solution containing 5 mM EDTA, 150 mM NaCl, 50 mM Tris-HCl (pH 8.0), and a cocktail of phosphatase and protease inhibitors (Roche Molecular Biochemicals). The samples were separated by SDS-PAGE electrophoresis and transferred to PVDF membranes. The immunoblots were developed using the following antibodies (Cell Signaling): rabbit polyclonal anti-PARP, mouse monoclonal anti-caspase 3, mouse monoclonal anti-caspase 8, mouse monoclonal anti-caspase 9, mouse monoclonal anti-p21, mouse monoclonal anti-p53, rabbit polyclonal anti-AKT, rabbit monoclonal anti-phospho-AKT (Ser473), rabbit monoclonal anti-mTOR, rabbit monoclonal anti-phospho-mTOR, rabbit monoclonal anti-cyclin B1, rabbit anti-phospho-cdc2 (Tyr15), rabbit anti-cdc2, rabbit anti-phospho-cdc25C (Ser216), and rabbit anti-cdc25C. Mouse anti-cyclin D1 antibody was obtained from Santa Cruz Biotechnology.

### Xenograft Mouse Models

The Nalm-6 (pre-B ALL) cells were transduced with retrovirus generated by the co-transfection of 293T cells with the retroviral construct GFP-FFluc and the psi-amphotropic envelope. Transduced tumor cells, which were marked by GFP expression, were selected by fluorescence-activated cell sorting (Nalm-6-FFLuc). To induce leukemia in a xenograft mouse model of systemic disease, 0.25×10^6^ cells were injected via the tail veins of NOD/SCID mice (Jackson Lab). Twenty-four hours later, the mice received daily intraperitoneal injections with 2 mg SF or vehicle and were monitored for leukemic cell expansion by bioluminescence detection each week. To induce subcutaneous tumors, 5×10^6^ Nalm-6-FFluc cells embedded in high concentration basement membrane matrix (BD Bioscience) were injected subcutaneously into the flanks of NOD/SCID mice. Bioluminescent imaging was performed after one week of tumor establishment (day 0 treatment) and after 4 and 7 days of SF treatment by oral gavage (2 mg, twice daily). For bioluminescence detection, the images were acquired in anesthetized mice using the IVIS Imaging System (Xenogen) 10 minutes after intraperitoneal injection with 50 mg/kg D-luciferin. All mice were maintained in specific pathogen-free conditions at the Baylor College of Medicine (Houston, TX, USA). All experiments were performed with the approval of the Institutional Animal Care and Usage Committee of Baylor College of Medicine.

### Statistical Analysis

Two-tailed unpaired Student’s *t*-tests were performed using GraphPad software. Differences were considered significant when the *P* value was <0.05. The statistics are indicated in each figure legend.

## Results

### Sulforaphane Induces Apoptosis in Pre-B ALL and T-ALL Cells

**Figure 1:**
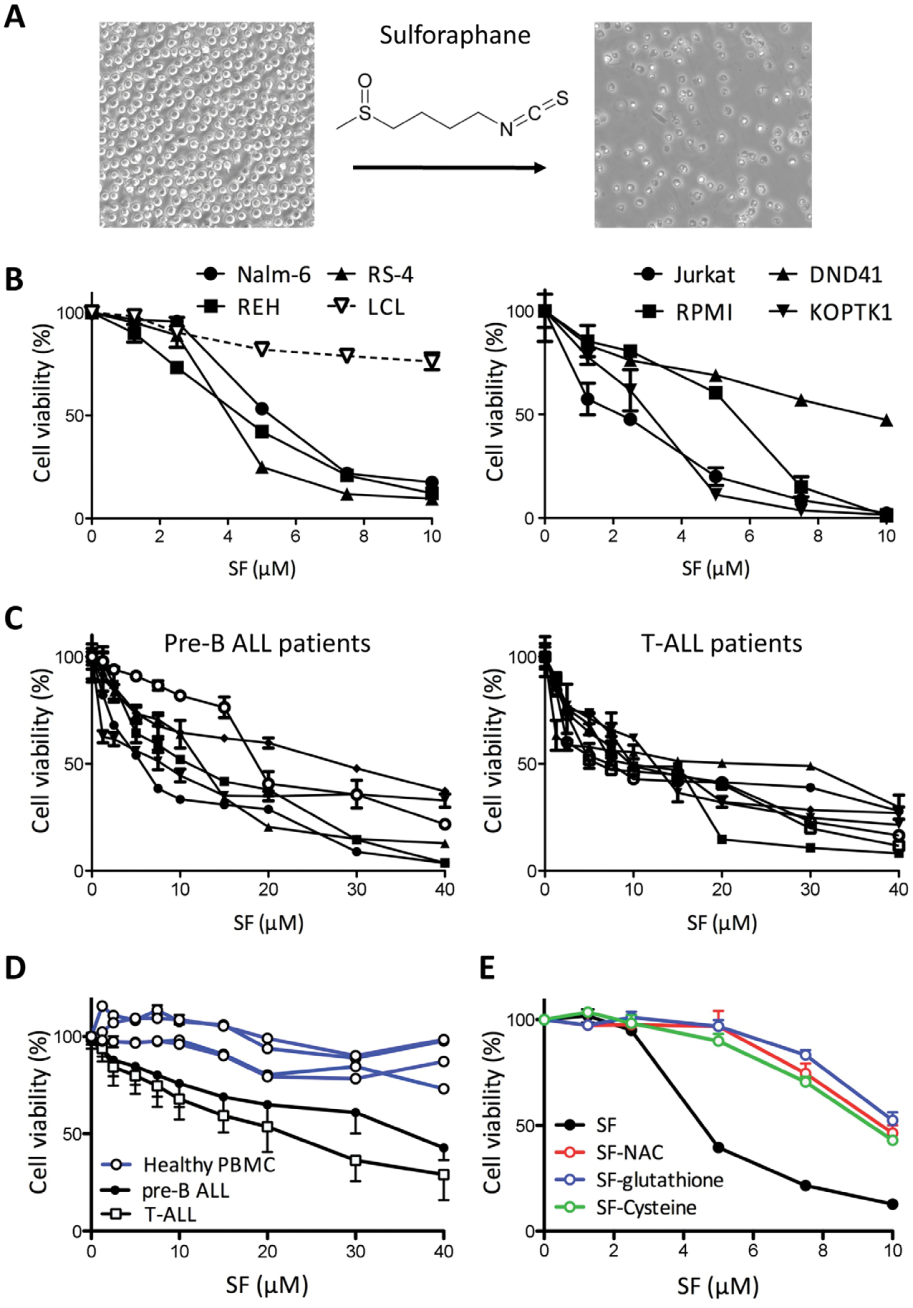
Inhibition of acute lymphoblastic leukemic cell survival by the SF isothiocyanate. (A) Phase contrast images of Nalm-6 cells after culture with 10 µM SF for 24 hours. The chemical structure of sulforaphane is shown. (B) Pre-B ALL cells (Nalm-6, REH and RS-4), non-leukemic LCL cells, and T-ALL cells (Jurkat, RPMI, DND41, and KOPTK1) were incubated with SF or vehicle for 48 hours (n = 3). Cytotoxicity was measured using an ATP-based cell viability assay and expressed as a percentage of the vehicle control. The data are representative of three independent experiments (mean and standard deviation). (C) Samples from pediatric patients diagnosed with pre-B ALL (n = 8) and T-ALL (n = 7) were incubated with the specified SF concentrations for 48 hours. Each patient sample was processed in triplicate (mean and standard deviation). (D) The effect of SF (24 h) on PBMC freshly isolated from healthy donors (n = 4, each measured in triplicates) was compared to cell viability in the pre-B and T-ALL patient samples (mean ± S.D.). (E) Nalm-6 cells were cultured in the presence of SF-glutathione, SF-NAC, SF-cysteine, or SF for 48 hours. Cell viability is shown as mean and S.D. (n = 3).

SF is a natural product found in cruciferous vegetables and has shown anti-cancer properties in breast, prostate, and colon cancer [33], [34], [35]. In this work, we investigated whether purified SF is also effective against hematological malignancies by testing its activity in ALL leukemic cells. We initially observed that SF (10 µM) significantly reduced the viability of the Nalm-6 human pre-B ALL cell line (Fig. 1A). We next examined the effects of different concentrations of SF (0–10 µM) on the viability of the cultured pre-B ALL and T-ALL leukemic cell lines compared to the EBV-transformed B-cell lymphoblastoid cell line (LCL) used as the “non-leukemic” control. SF selectively killed the Nalm-6, REH, and RS-4 pre-B ALL cells in a dose-dependent manner (Fig. 1B). In contrast, the incubation of non-leukemic cells with the highest concentration of SF resulted in a 20% reduction in cell viability (Fig. 1B). Similar to the pre-B ALL cells, the Jurkat, RPMI, DND41, and KOPTK1 T-ALL cell lines were susceptible to SF-mediated cytotoxicity, and nearly no viable leukemic cells survived after incubation with 10 µM SF (Fig. 1B). Consistent with the inhibitory concentrations reported for other cancer cells [29], [36], [37], SF exhibited a half-maximal inhibitory concentration (IC50) in the 1–7 µM range (Table 1). Because patient-derived cells often accumulate additional genetic aberrations during the establishment of a cell line, we further confirmed our findings using primary lymphoblasts collected at diagnosis from the pre-B ALL and T-ALL pediatric patients at the Texas Children’s Cancer and Hematology Centers. The patient lymphoblasts showed a dose-dependent cytotoxicity with an IC50 (48 h) in the 7–13 µM range, and only one of six pre-B ALL samples displayed a poor response to SF (Fig. 1C, Table 1). Collectively, our data demonstrate that SF significantly reduced the cell viability of established ALL cell lines and patient samples. The next question was to examine the toxicity of SF on normal peripheral blood mononuclear cells (PBMC). We cultured PBMC freshly isolated from 4 different healthy donors in the presence of SF (0–40 µM) for 24 hours, which is the time reported for complete elimination of SF from blood [37]. In contrast to lymphoblasts from ALL patients, normal PBMC were less susceptible to SF showing 10–20% cytotoxicity with the highest concentration (Fig. 1D). Even though longer incubations (48 hours) reduced survival of normal PBMC due to their limited proliferative and survival capacity *in vitro*, the IC50 (30.2±7.2 µM, n = 4) was significantly higher (p<0.001) than primary samples from pre-B ALL (13.1±8.1 µM, n = 6) and T-ALL (10.1±2.0 µM, n = 7) patients. Finally, we investigated whether the metabolism of SF leads to abrogation of its cytotoxic properties, which is particularly important for future translation into the clinic. In contrast to SF (IC50 4.7 µM), the survival of Nalm-6 cells cultured in the presence of different doses of SF-glutathione, SF-cysteine or SF-N-acetyl-cysteine was significantly improved, with IC50s 9 µM (Fig. 1E). For example, 7.5 µM of SF-metabolites induced only 20% cytotoxicity after 48 hours of incubation compared to 80% for same concentration of SF. Thus, future clinical trials should aim at maintaining a therapeutic concentration of SF (active form).

**Table 1:** IC50 values in leukemic cell lines, primary lymphoblast cells from pre-B ALL and T-ALL patients.

Cells	IC50 (µM) (24 h)	IC50 (µM) (48 h)
LCL	80.81 (26.80–243.60)	30.83 (17.67–53.80)
Nalm-6	8.91 (7.43–10.83)	4.58 (4.37–4.79)
REH	10.26 (9.40–11.20)	4.38 (4.10–4.67)
RS-4	9.86 (9.35–10.41)	3.85 (3.69–4.01)
Jurkat	4.72 (4.19–5.25)	2.39 (1.44–3.34)
RPMI	7.87 (6.40–9.34)	5.72 (4.12–7.32)
DND41	13.49 (12.84–14.15)	7.14 (6.27–8.01)
KOPTK1	3.04 (2.16–3.92)	1.24 (0.84–1.66)
preB-1	22.02 (19.39–25.00)	5.46 (4.91–6.07)
preB-2	59.53 (45.00–78.73)	10.09 (9.02–11.29)
preB-3	36.30 (32.19–40–94)	10.98 (9.94–12.13)
preB-4	21.00 (18.13–24.32)	6.21 (5.17–7.45)
preB-5	51.29 (39.81–66.09)	25.36 (22.43–28.68)
preB-6	331.2 (211–519.5)	20.81 (22.59)
T-1	51.52 (38.04–69.77)	11.64 (10.87–12.47)
T-2	20.28 (18.48–22.24)	9.55 (8.31–11.0)
T-3	50.39 (37.33–68.04)	13.02 (9.11–18.63)
T-4	22.46 (19.72–25.58)	11.36 (10.32–12.49)
T-5	9.12 (7.97–10.45)	10.42 (9.31–11.67)
T-6	16.95 (15.50–18.54)	7.12 (6.11–8.32)
T-7	21.32 (19.52–23.28)	8.01 (6.92–9.24)

* The IC50 values were obtained by triplicate from each sample (pre-B and T-ALL). The values in parentheses represent the 95% confidence interval.

**Figure 2:**
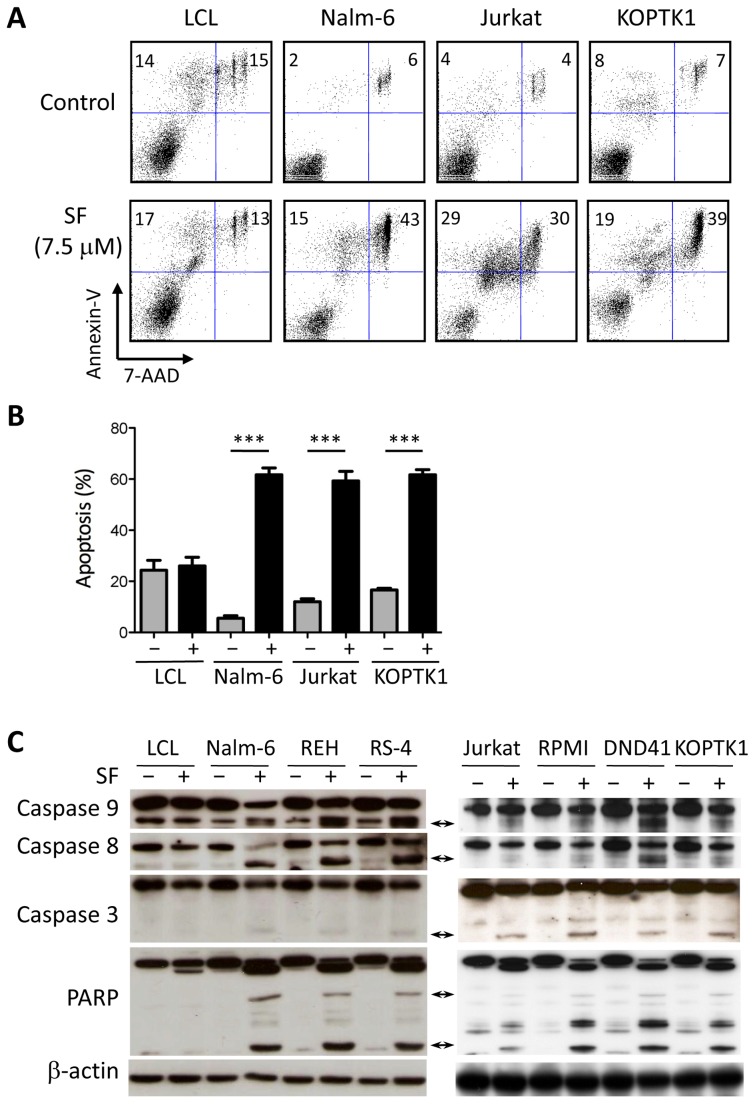
SF induces apoptosis selectively in ALL cell lines. (A) Apoptosis was evaluated by annexin-V and 7-AAD staining of LCL, Nalm-6, Jurkat and KOPTK1 cells cultured in the absence or presence of 7.5 µM SF. The data are representative of three independent experiments. (B) The percentages of annexin-V-positive cells were determined for each cell line cultured in the presence or absence of SF. (C) SF activates the proteolytic cascade of caspases and PARP in leukemic cells. LCL cells (control), pre-B ALL cells (Nalm-6, REH, and RS-4), and T-ALL cells (Jurkat, RPMI, DND41, and KOPTK1) were incubated with 7.5 µM SF for 24 hours and analyzed by immunoblotting. The arrows indicate the cleaved forms of the caspases and PARP. β-actin was used as a loading control. The data represent the mean and standard deviation (n = 3). *** *P*<0.001 (two-tailed Student’s *t*-test).

The reduction of cell viability indicates that SF induced cell death in leukemic cells. To test this hypothesis, we incubated ALL and LCL cells with 7.5 µM SF for 48 hours and then stained the cells with annexin-V and 7-AAD for flow cytometric analysis. In contrast to the LCL cells, SF induced apoptosis in the Nalm-6, Jurkat and KOPTK1 cells (Fig. 2A,B). Selectivity for leukemic cells is a desired property of chemotherapeutic agents. Next, we analyzed the effect of SF on the cleavage of caspases to elucidate the mechanism of SF-induced cell death in ALL cells. Pro-apoptotic signals activate caspases 8 and 9, which lead to the activation of the effector caspase 3. No significant cleavage of caspases was observed in LCL cells treated with SF (Fig. 2C). In contrast, the pre-B ALL and T-ALL cell lines showed cleavage of caspases 3, 8, and 9 (Fig. 2C). The inactivating cleavage of the DNA repair enzyme PARP was also observed only in ALL cells treated with SF (Fig. 2C).

### Sulforaphane Induces G2/M Cell-cycle Arrest and Inhibits AKT-mediated Survival Signals in ALL Cells

**Figure 3:**
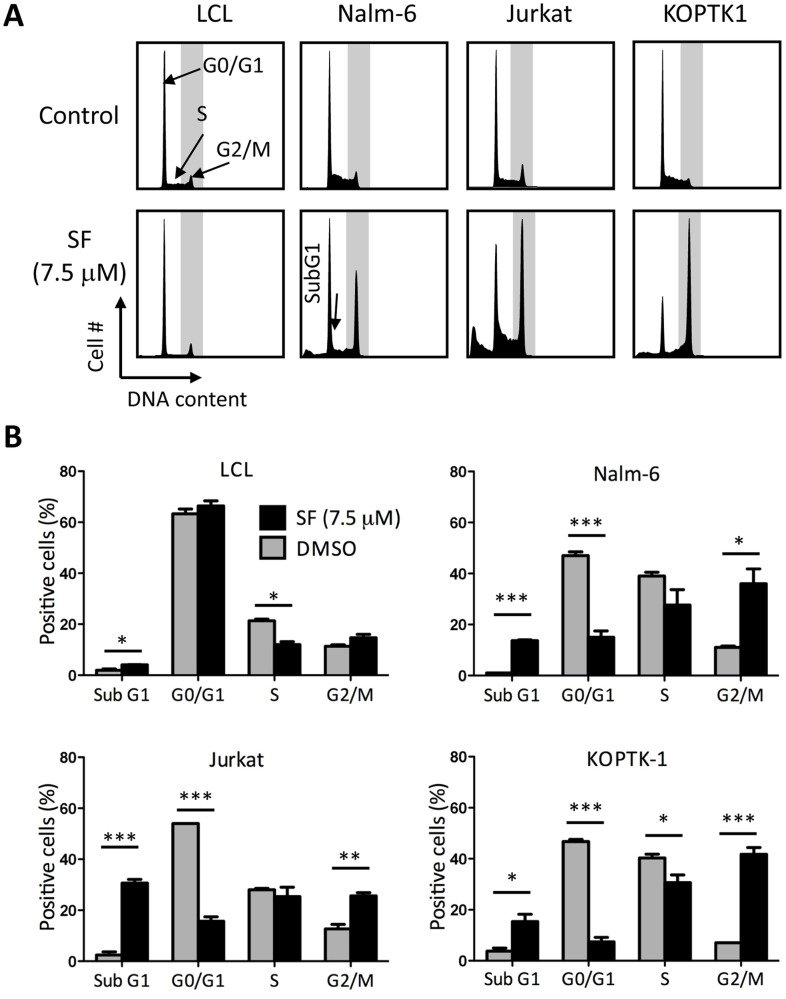
SF causes G2/M cell cycle arrest in leukemic cells. (A) The DNA content of LCL, Nalm-6, Jurkat and KOPTK1 cells incubated with 7.5 µM SF or vehicle for 24 hours was analyzed by propidium iodide staining of nuclei and flow cytometry. A representative profile is shown. (B) The cell cycle distribution was calculated as described in the Methods. The data represent the mean and standard deviation (n = 3). * *P*<0.05, ** *P*<0.01, *** *P*<0.001 (two-tailed Student’s *t*-test).

Chemical compounds can induce cytotoxicity by causing aberrations in the cell cycle checkpoints, thus forcing cells to undergo division and cell death. To study the effect of SF on the cell cycle, we examined the DNA content of LCL, Nalm-6, Jurkat and KOPTK1 cells incubated with 7.5 µM SF by flow cytometry. Interestingly, SF-treated ALL cells accumulated in the G2/M and sub-G1 phases of the cell cycle, while LCL cells showed lower percentages in S phase and a slight increase in apoptosis (Fig. 3A). The aggregate analysis clearly demonstrates that the leukemic cells treated with SF accumulated in the G2/M and sub-G1 phases with a concurrent reduction in the G0/G1 and S phases (Fig. 3B).

**Figure 4:**
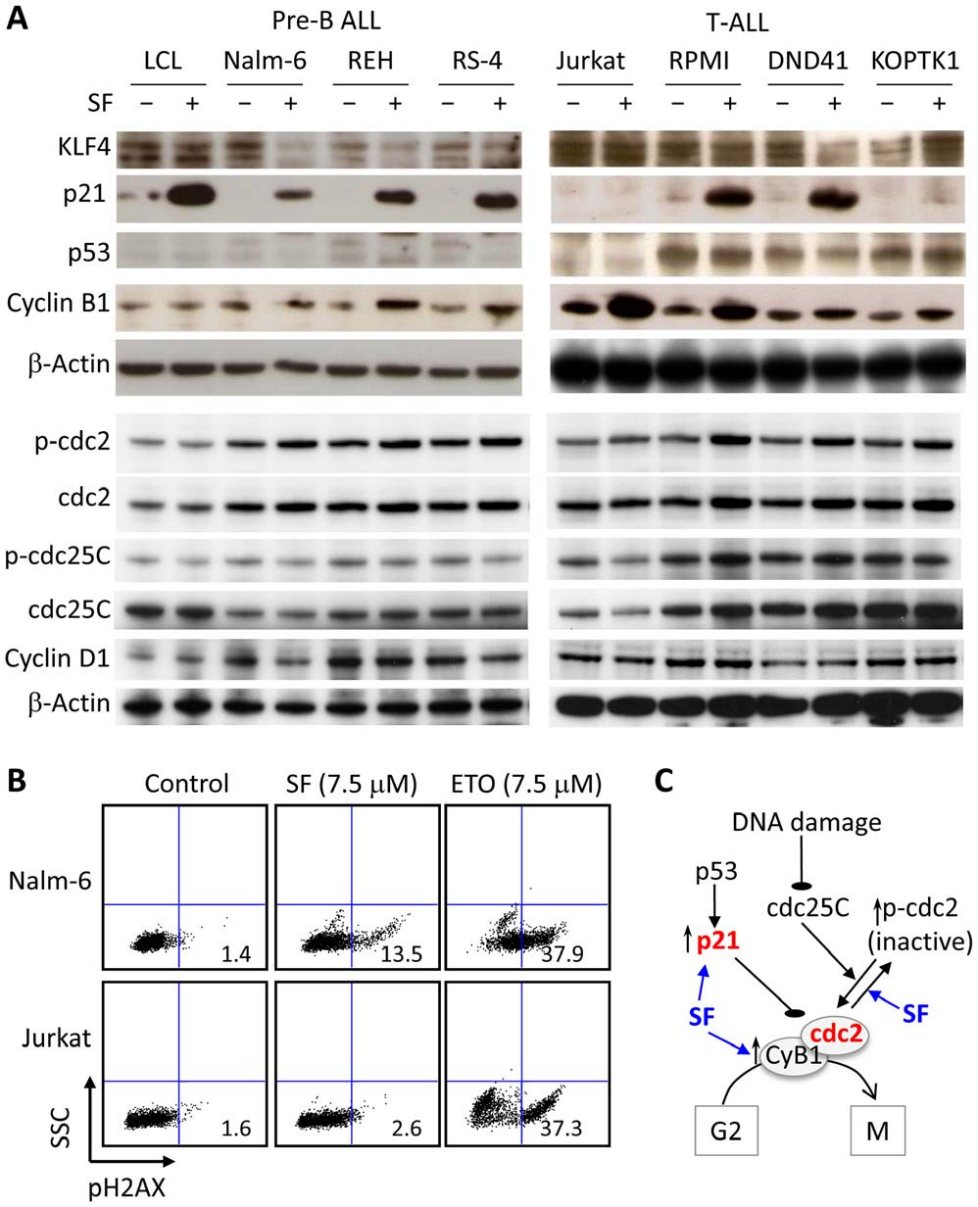
Effect of SF on the levels of total and phosphorylated proteins involved in the G2/M cell cycle arrest. (A) The protein levels of KLF4, p21, p53, cyclin B1, cyclin D1, cdc2, and cdc25C and phosphorylation of cdc2 (Tyr 15) and cdc25C (Ser 216) were evaluated by immunoblots in LCL, pre-B ALL, and T-ALL cell lines incubated with 7.5 µM SF or vehicle for 24 hours. β-actin was used as a loading control. The data are representative of three independent experiments. (B) Detection of phospho-H2AX (pH2AX) in nuclei by flow cytometry in Nalm-6 and Jurkat cells treated with 7.5 µM of SF or Etoposide (ETO) for 24 hours. The data are representative of three independent experiments. (C) Diagram depicting the role of SF in the G2/M cell cycle arrest of ALL cells.

To gain further insight into the mechanism of SF, we determined the expression of cell cycle regulators and pro-survival signals. Cell lysates from pre-B ALL cells (Nalm-6, REH, RS-4), T-ALL cells (Jurkat, RPMI, DND41 and KOPTK1), and control LCL cells were incubated for 24 hours in the presence of 7.5 µM SF or vehicle control and then analyzed by immunoblotting. We measured the expression of the tumor suppressor KLF4 and the cell cycle inhibitor p21 because SF was shown to induce the expression of both proteins in Caco-2 cells [29]. Consistent with a previous report [38], SF increased the levels of p21 independently of p53 in most ALL cells, except the Jurkat and KOPTK1 cells (Fig. 4A). In LCL cells, p21 expression was associated with the inhibition of proliferation rather than the induction of cell death (Figs. 3, 4). The low levels of KLF4 expression observed in ALL cells, regardless of SF incubation, suggests that the upregulation of p21 was independent of KLF4 activity (Fig. 4A). Epigenetic silencing of the KLF4 gene, an alternate mechanism that may be responsible for the low expression of KLF4 in ALL cells, was supported by the 8-fold (KOPTK1), 13-fold (RMPI), 6-fold (DND41), and 5-fold (Jurkat) increases of KLF4 expression upon culture of the leukemic cells in the presence of 5-azacytidine (not shown). In accordance with our findings, KLF4 gene was found hypermethylated in adult T-ALL [39]. We also measured Cyclin D1 levels because SF can induce G1 cell cycle arrest by inhibiting expression of Cyclin D1 in human colon carcinoma HT-29 cells [38]. No significant differences were observed upon SF treatment, only pre-B ALL cells showed a modest reduction in Cyclin D1 expression likely due to the G2/M arrest (Fig. 4A).

To further study the mechanism of cell cycle arrest, we first analyzed the levels of Cyclin B1 in SF-treated ALL cell lines because the Cdc2-Cyclin B1 complex controls the G2/M transition. It has been reported that SF induces G2/M arrest by altering the levels of Cyclin B1 in PC-3 and LnCap prostate cancer cell lines [28], [40]. In acute lymphoblastic leukemia, SF increased the expression of Cyclin B1 in the REH, RS-4 and T-ALL cell lines (Fig. 4A). Immunoblots revealed increased levels of phospho-Cdc2 (Tyr-15) in ALL cells treated with SF, suggesting that SF inhibits the Cdc2/Cyclin B1 complex by increasing phosphorylation of Cdc2 (inactive) despite elevated levels of Cyclin B1 (Fig. 4A). Cdc25C activates Cdc2/Cyclin B1 complex by dephosphorylating Cdc2 and it is inactivated by phosphorylation at Ser 216 in response to DNA damage [41], [42]. Since we did not detect significant changes in total and phosphorylated Cdc25C, we concluded that SF likely activates the Wee1 or Myt1 kinases that phosphorylate Cdc2 [43], [44], in addition to the inhibitory role of p21 in the G2/M transition. To investigate whether SF causes DNA damage, we determined by flow cytometry nuclear expression of phospho-H2AX, a sensitive marker of double-stranded DNA breaks, using etoposide (topoisomerase inhibitor) as a positive control. Nalm-6 cells treated with 7.5 µM SF showed considerably lower phospho-H2AX content compared to Nalm-6 cells treated with etoposide, while almost no positive cells were detected in Jurkat cells (Fig. 4B). Collectively, our data indicate that SF induces G2/M arrest by inactivating Cdc2 and elevating p21 levels without inducing significant DNA damage (Fig. 4C).

**Figure 5:**
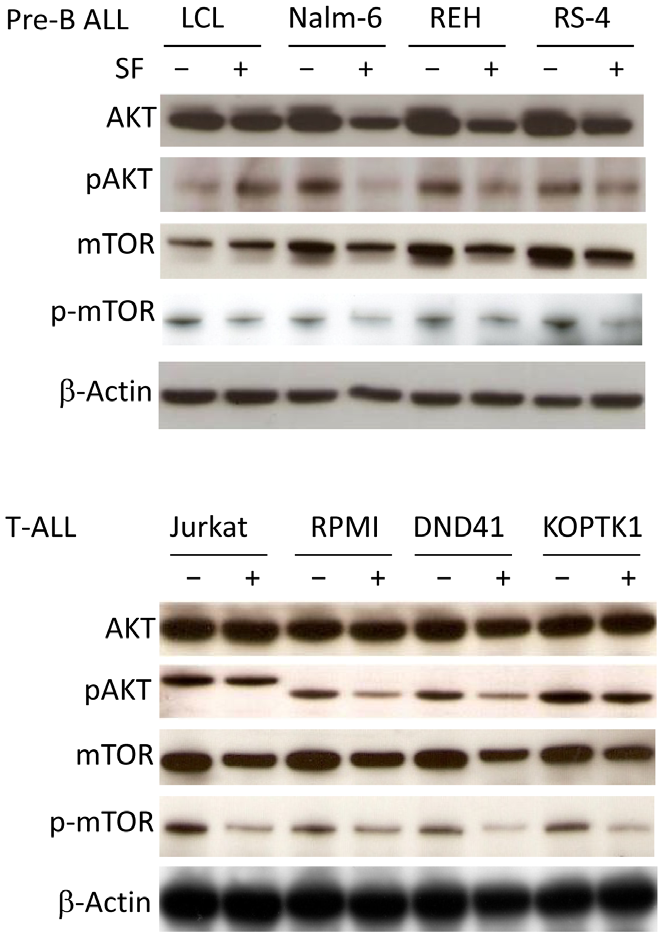
SF inhibits the AKT/mTOR survival pathway in leukemic cells. The protein levels of phosphorylated and total AKT and mTOR were examined by immunoblotting. LCL, pre-B ALL, and T-ALL cell lines were incubated with 7.5 µM SF or vehicle for 24 hours. β-actin was used as a loading control. The data are representative of three independent experiments.

Finally, we were interested to study the effect of SF on survival signals, as many patients with T-ALL exhibit activation of the AKT pathway. Interestingly, SF inhibited the AKT and mTOR pathways by lowering the protein levels of both total and phosphorylated AKT and mTOR (Fig. 5). In contrast to the pre-B and T-ALL cell lines, the LCL cells showed a slight activation of AKT signaling in response to SF treatment. Collectively, these findings indicate that SF can inhibit AKT/mTOR-mediated survival signals in leukemic cells.

### Pre-clinical Testing of SF Using Xenograft Leukemia Models

**Figure 6:**
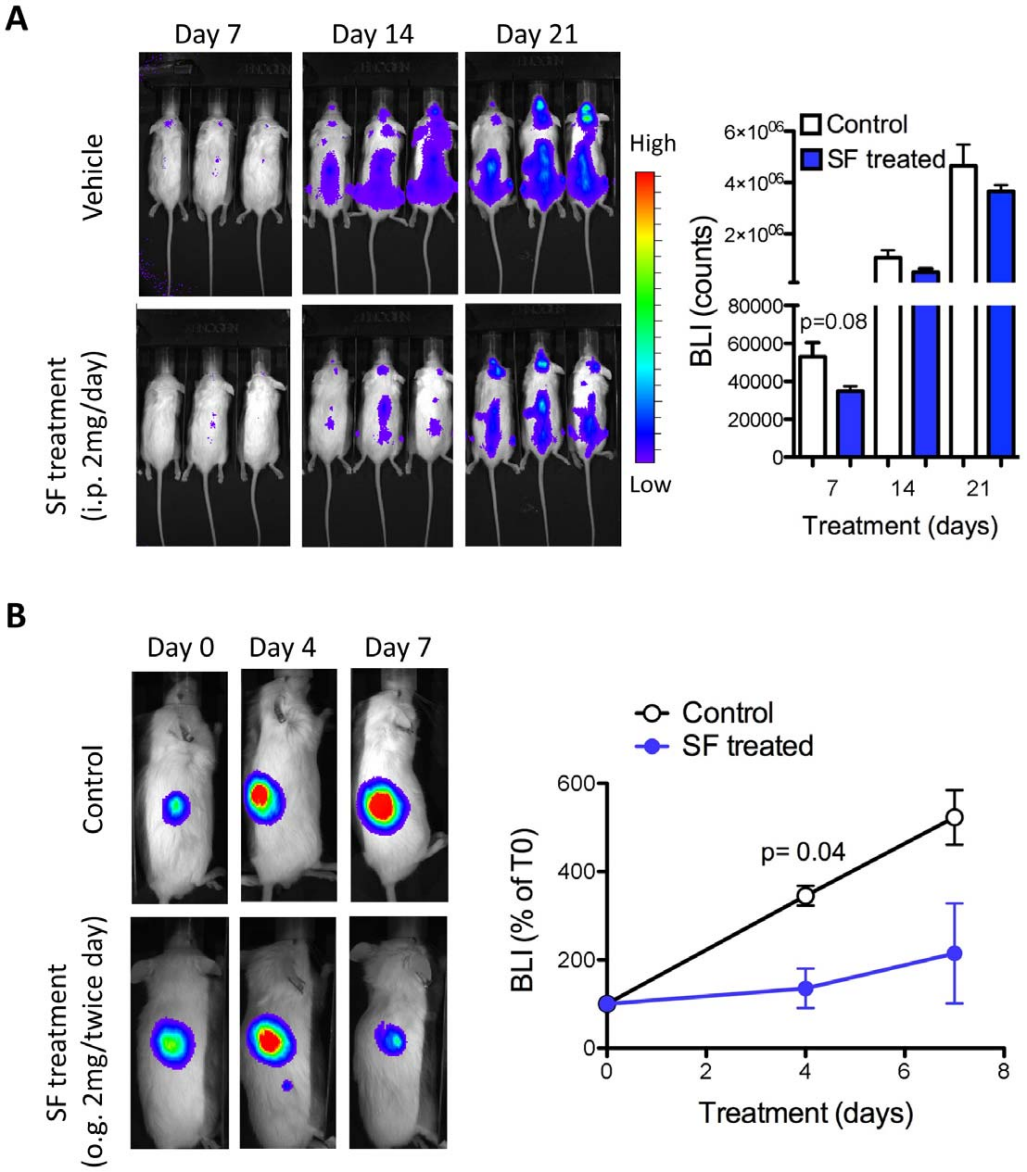
*In vivo* anti-leukemic effect of SF in ALL xenograft models. (A) Nalm-6 cells were transduced with the FFluc retrovirus and injected intravenously into NOD/SCID mice. Twenty-four hours later, the mice were treated with SF or vehicle (2 mg i.p. daily) and monitored weekly for the distribution of leukemic cells by bioluminescence imaging (BLI). (B) The Nalm-6-FFluc cells were mixed with a high-protein matrigel and injected into the flank of NOD/SCID mice. One week later, tumor establishment was confirmed by BLI; then, the mice were treated by oral gavage (2 mg/gave, twice daily) for 7 days. The total counts were determined for the control- and SF-treated mice. The data are representative of three independent experiments. The statistical significance was calculated using the two-tailed Student’s *t*-test.

First, we tested the efficacy of SF in controlling the expansion of Nalm-6 cells injected in NOD/SCID mice to model systemic disease. The leukemic cells were detected by bioluminescence in the first week whereas in the peripheral blood were detected by flow cytometry at a later stage, when mice showed signs of disease (weight loss and hind-limb paralysis). The NOD/SCID mice injected with Nalm-6-FFluc cells were randomized to two groups: the experimental group received daily administration of SF (2 mg, i.p.), and the control group received vehicle starting 24 hours after injection of the leukemic cells. Bioluminescent imaging of the tumor-bearing mice showed that treatment with SF reduced disease progression in the first 2 weeks, although we did not observe significant differences in survival (Fig. 6A). The partial response may be due to the low SF concentration in tissues, such as the bone and spine, where leukemic cells initially reside and expand during the first few weeks. Therefore, we next tested the oral administration of SF in pre-established subcutaneous xenograft tumors. Mice that received the vehicle showed a 4-6-fold increase in tumor burden compared to the tumor size before treatment (day 0). In contrast, the mice treated with SF showed a significant reduction in tumor growth after 4 days of treatment (Fig. 6B). Our findings demonstrate the effectiveness of SF in controlling the expansion of human leukemic cells *in vivo*. The low toxicity associated with the administration of extracts enriched in SF in human clinical trials supports further studies on the role of SF in hematological malignancies.

## Discussion

There is emerging evidence that SF has anti-carcinogenic properties in addition to the chemopreventive effect of broccoli extracts that have been extensively investigated in solid tumors. The main goal of this study was to test the anti-leukemic properties of SF in the most prevalent hematological cancer in children because the development of new chemodrugs for pediatric patients has lagged behind the development of new therapies for adult leukemia. In this work, we found that synthesized DL-Sulforaphane induced a dose-dependent cytotoxicity in ALL cell lines and primary lymphoblasts from pediatric patients diagnosed with pre-B ALL or T-ALL. Most importantly, normal peripheral blood mononuclear cells were more resistant than patient lymphoblasts to SF mediated cytotoxicity.

Based on previous reports indicating that SF-mediated cytotoxicity in Caco-2 cells was associated with the increased expression of the tumor suppressor KLF4 and the cyclin-dependent kinase inhibitor p21, we initially hypothesized that SF would also induce KLF4 expression in ALL cells [29]. However, the low levels of KLF4 observed in ALL cell lines suggested an epigenetic silencing that could not be reversed by SF; in support of this hypothesis, we observed increased KLF4 expression in T-ALL cell lines cultured with a demethylating agent. Despite the low expression levels of KLF4 and p53, we observed a significant upregulation of p21 in pre-B ALL and T-ALL cells following SF treatment. Because the anti-tumor activity of many conventional chemotherapeutic agents (e.g., doxorubicin, cisplatin, and etoposide) is mediated by p53 [45], [46], the p53-independent activation of p21 that was observed upon SF treatment could represent an alternative therapeutic approach for ALL patients that fail to respond to conventional agents targeting p53. In addition to p21 upregulation, SF inactivated the Cyclin B1 and Cdc2 complex by promoting phosphorylation of Cdc2 in spite of increased levels of Cyclin B1. Our findings are consistent with previous reports showing that SF induces G1 or G2/M cell cycle arrest by regulating cell cycle proteins [26], [27], [28], [29], [47].

Several reports have shown activation of the mitochondrial or death receptor pathways in cancer cells cultured with SF [21], [22], [23], [28], [48]. In this work, we showed that SF induced apoptosis in pre-B ALL (Nalm-6, REH, and RS-4) and T-ALL (Jurkat, RPMI, DND41, and KOPTK1) cell lines by activating caspases 3, 8, and 9 and inactivating PARP. Interestingly, SF-mediated cytotoxicity appeared specific to leukemic cells, as no significant activation of the caspase proteolytic cascade was observed in the non-leukemic control cells. In addition to cell cycle arrest and apoptosis, SF inhibited the AKT/mTOR pathway in ALL cells by lowering the expression of both the total and phosphorylated forms of AKT and mTOR. This result was of interest because the AKT/mTOR survival pathway is the target of choice in the development of alternative ALL therapies since approximately 30% of pre-B ALL patients display mutations in the signaling molecules that activate AKT, including RAS, PTPN11 and FLT3 [49], [50], [51], [52]. In addition, a gene expression study showed that AKT activation was associated with resistance to glucocorticoids in pre-B ALL [10]. Inactivation of PTEN by deletion, mutation, epigenetic silencing, or post-translational inactivation has been reported in T-ALL patients, suggesting that activation of the AKT pathway also contributes to the development of T-ALL [13], [53] [54]. Taken together, our data indicate that SF may be effective in the treatment of ALL patients with activated AKT pathways.

Several therapeutic compounds that target the cell cycle and the PI3K/AKT/mTOR pathway are being tested as ALL therapies. For example, clofarabine and nelarabine are cell-cycle dependent CDK inhibitors that have been recently approved to treat pediatric ALL [55]. Alternatively, several PI3K inhibitors with distinct spectra of activity are in clinical development as combination therapies to eliminate drug-resistant ALL, such as the dual PI3K/mTOR inhibitor NVP-BEZ235 and the PI3K/PDK-1 inhibitor BAG956 [56], [57]. The PI3K-AKT inhibitor Ly294002 has also shown marked activity in leukemia cells [58]. Our study showed that, in addition to perturbing the cell cycle, SF also inhibited the AKT survival pathway by lowering protein levels of total and phosphorylated AKT and mTOR. There are several advantages to using natural food products for cancer treatment such as correct chirality to bind biomolecules, safety, and the record of the natural products tested in clinical trials. SF also induced phase 2 metabolic enzymes (detoxification) in addition to its anti-carcinogenic properties [19], [59]. Therefore, SF may perform dual activities as a chemotherapeutic agent by inducing cell death in cancer cells and reducing toxicity in multi-drug intensified treatments.

The pharmacokinetics of SF have been extensively studied in humans. After oral intake of broccoli, SF reaches a transient plasma concentration of approximately 2 µM [60], [61]. The bioavailability of SF depends on food processing, as cooked broccoli demonstrated lower serum concentrations compared to raw broccoli [62]. Inhibitory concentrations in the micromolar range (1–7 µM in ALL cell lines and 5–20 µM in primary lymphoblastic cells) indicate that a therapeutic concentration of SF cannot be reached through the dietary consumption of cruciferous vegetables. We tested the efficacy of SF in two pre-clinical xenograft models of ALL as a proof-of-principle that SF can inhibit the expansion of leukemic cells *in vivo*. Intraperitoneal administration of SF to NOD/SCID mice injected with Nalm-6 reduced the expansion of leukemic cells in the first two weeks. The inability to efficiently eliminate or control the expansion of leukemic cells *in vivo* was likely due to the low concentrations achieved in leukemic cell niches such as the bone marrow. However, oral administration of SF led to a significant reduction in the expansion of leukemic cells in a model for pre-established lymphoma tumors. Collectively, our pre-clinical studies showed that SF can inhibit the expansion of leukemic cells *in vivo*, which supports future clinical trials as adjunctive agent.

In summary, the results presented in this study demonstrate the anti-leukemic properties of SF in acute lymphoblastic leukemia by inducing cell cycle arrest and apoptosis. Furthermore, we showed that SF inhibits the AKT/mTOR survival pathway. This is the first report on the anti-leukemic property of SF in hematological malignancies, supporting its use as an adjunctive chemotherapy in ALL.
